# Ultrasonographic and elastographic biometry in adult major salivary glands: a preliminary case-control report

**DOI:** 10.1038/s41598-019-45230-y

**Published:** 2019-06-20

**Authors:** Kai-Min Fang, Ming-Hsun Wen, Wan-Lun Hsu, Chih-Ming Chang, Pei-Yu Hou, Li-Jen Liao

**Affiliations:** 1 Graduate Institute of Basic Medical Science, College of Medicine, China Medical University, Taichung, Taiwan; 2 Department of Otolaryngology, Far Eastern Memorial Hospital, New Taipei City, Taiwan; 3 Genomics Research Center, Academia Sinica, Taipei, Taiwan; 4 Department of Radiation Oncology, Far Eastern Memorial Hospital, New Taipei City, Taiwan; 5 Department of Electrical Engineering, Yuan Ze University, Taoyuan, Taiwan; 6 Department of Clinical Engineering, Far Eastern Memorial Hospital, New Taipei City, Taiwan

**Keywords:** Saliva, Salivary gland diseases

## Abstract

Specifications about the size and stiffness of healthy salivary glands with ultrasound (US) are not available for Asian people. Using a Toshiba Apolio 500 US platform, we determined the size (including anterior-posterior median length, median paramandibular depth dimension, and cranio-caudal height) and hardness of 100 healthy submandibular and parotid glands in volunteers without a history of disease affecting the salivary glands or post-radiation, and compared the dimensions to those of 36 parotid glands and 37 submandibular glands in post-irradiated patients. The dimensions of the parotid and submandibular glands were significantly correlated with body weight. However, the dimension of the parotid glands was not significantly correlated with that of patients with prior radiation; the shear wave velocity (SWV) significantly increased (1.99 m/s versus 2.43 m/s, p-value < 0.01). The dimension of the submandibular glands was significantly correlated with prior radiation, where the SWV also significantly increased (2.32 m/s versus 2.50 m/s, p-values < 0.01). We find that US is a useful tool for assessment of the reference dimensions and hardness of major salivary glands that may be altered by irradiation.

## Introduction

An increasing number of head and neck (H&N) surgeons and otolaryngologists use ultrasound (US) to check patients’ necks. For H&N surgeons, mostly, they use US to examine neck masses, such as lymphadenopathy^1,2^, thyroid nodule^3^, and salivary tumor assessment^4,5^. In addition, real-time US can guide biopsy procedures^6^. To date, very few studies have focused on the biometry and physiology of salivary glands with US^7–9^.

The salivary glands are composed of three major salivary (parotid, submandibular and sublingual) and minor salivary glands. It is easier to investigate the parotid and submandibular (SM) glands by US as a result of encapsulation than the sublingual and minor salivary glands. Disease conditions, such as inflammatory or neoplastic condition, will lead to enlargement of the salivary glands, while sclerosing disease will lead to atrophy of the glands. Therefore, it is important to develop a reference standard for US in order to diagnose salivary gland diseases. US could provide an easier and more feasible tool to check these conditions. Other factors related to the size and function of salivary glands have been reported, such as diabetes^10^, smoking^11^, hypertension^12^ and alcoholic beverage consumption^13^. These conditions all potentially have an impact on the size and function of salivary glands. Aging has also been reported to lead to atrophy^14^, fibrosis^15^ and accumulation of intercellular adipose tissue^16^ in salivary glands, which may further result in dry mouth in the elderly.

However, few previous studies have assessed the size of normal salivary glands with high resolution US^8^. Dost *et al*.^7,8^ reported the first biometry study on salivary glands. They reported that the dimensions of the parotid gland were correlated with body weight (BW), but those of the submandibular gland were not. Additionally, they found that age was not correlated with the dimensions of these two major salivary glands^8^.

Furthermore, quantitative elastography is a novel technique for examining the stiffness of the major salivary glands. In diseased salivary glands, such as primary Sjögren’s syndrome^17^ and post-irradiated glands^18^, the shear wave velocity (SWV) is higher than that in normal glands. However, these results varied as a result of the measurement site^19^ and pre-compression level^20^. Therefore, the related factors of the biometry and stiffness of the major salivary glands with US are not clear.

The aim of this study is to clarify the ultrasonographic and elastographic biometry of major salivary glands and elucidate possible related factors for those glands. We used both ultrasound and elastography to compare glandular biometry of healthy individuals and individuals who had previously received radiation therapy.

## Materials and Methods

**Figure 1:**
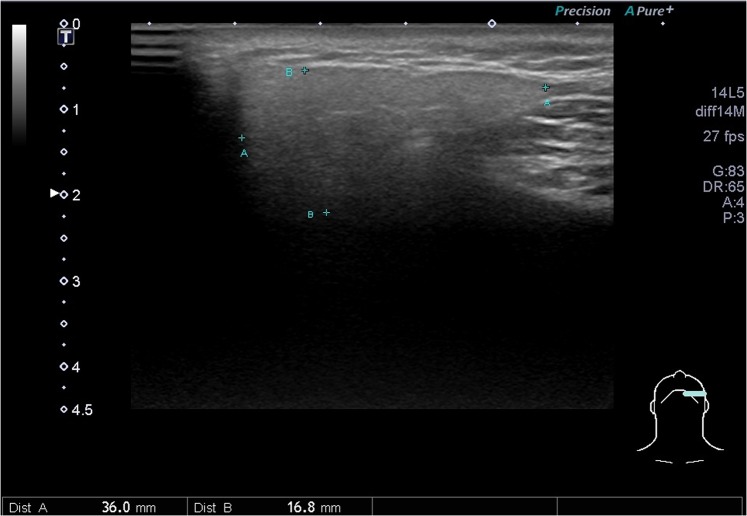
Dimensions of anteroposterior length (AA) and paramandibular depth (BB) of the submandibular gland.

**Figure 2:**
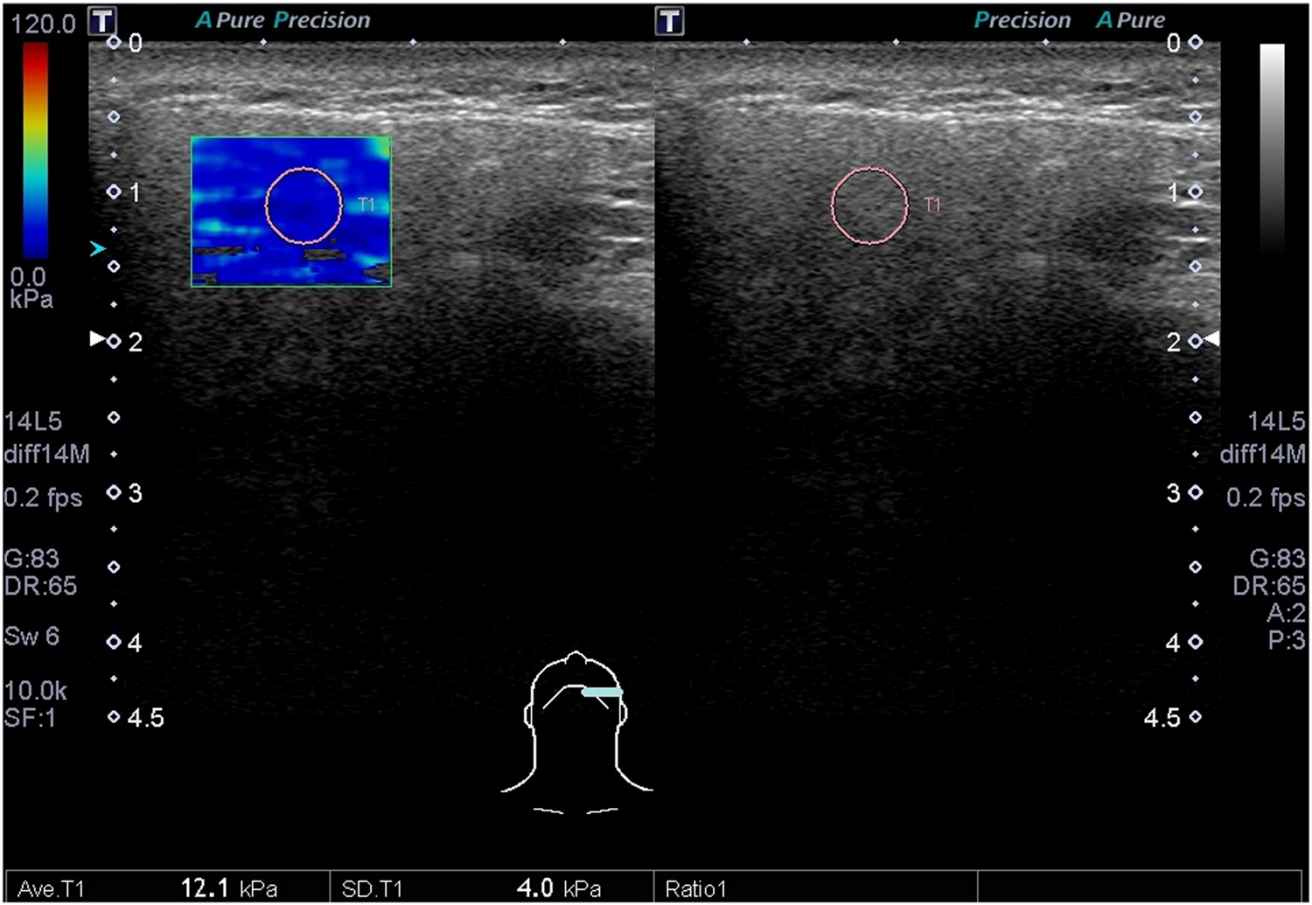
The SWV over the central part of the SM gland was recorded.

This study protocol was approved by the Far Eastern Memorial Research Ethics Review Committee (IRB No: 104180-E). The study was conducted in accordance with relevant guidelines and regulations. Informed consents were obtained from all participants. The sonograms were performed by L.J.,Liao (with experience of more than 10,000 ultrasound procedures), who was blind to the included cases, using a Toshiba Apolio 500 platform (Otawara, Japan) with a high-resolution 5-MHz to 14-MHz real-time linear-array transducer. All methods performed were in accordance with the manufacturer’s relevant guidelines and institutional regulations. We measured three dimensions of each submandibular (Fig. 1.) and parotid gland, including the dimensions of anterior-posterior length and paramandibular depths in the transverse axis and the dimensions of cranio-caudal height, as described in previous literature^7–9^. The ROI (region of interest, about 5 mm in circle) over the central part of the parotid and submandibular glands were recorded with mean SWV (Fig. 2.). From January 2016 to March 2018, a recruitment announcement for clinical trial was posted in our outpatient department of FEMH. Healthy adults (age ≧ 20) without cancer or major disease history, acute viral or bacterial inflammation were recruited as participants of this study, and were invited to receive salivary glands ultrasound measurements. During the period, we also collected data from patients who were referred from our hospital to our ultrasound center for head and neck examination. Subjects with head and neck cancer were included as part of the post-radiation group. We excluded patients without prior radiotherapy to the neck, or patients with ongoing radiotherapy or residual tumor. The radiotherapy of these patients was conducted in our hospital which consisted of three techniques: TOMO (Helical Tomotherapy), VMAT (Volumetric Modulated Arc Therapy) and IMRT (Intensity Modulation Radiation Therapy).We measured their body weight and body height before US examination. We also recorded the patients’ histories of diabetes, hyperlipidemia and hypertension according to their medical records.

### Statistical analysis

Continuous data were expressed as the mean (standard deviation) or median (interquartile range, IQR) where appropriate. Categorical data were expressed as a number and percentage. Medians were compared using the nonparametric M-W U test. A p-value < 0.05 was considered to indicate significance. The statistical analyses were performed using STATA software, version 12.0 (Stata Statistical Software: Release 12. College Station, TX: Stata Corp LP).

## Results

50 healthy patients from our outpatient department volunteered to join the study. A total of 3737 patients were referred to our department for head and neck examinations, including 427 patients with head and neck cancer, 143 patients of whom had received radiotherapy. We excluded patients with residual tumor or ongoing radiotherapy. 41 post-radiation head and neck cancer survivors were invited, 20 of whom agreed to join the study. In sum, a total of 70 patients participated in our study. Their age ranged from 20 to 89 years with a mean of 43 ± 15 years. The BMIs for these patients were 22.86 ± 3.74 and 25.60 ± 3.30 kg/m^2^ for healthy volunteers and post-radiation patients, respectively. The demographic data of the volunteers are summarized in Table 1. A total of 20 patients received radiation therapy to the neck, the median (interquartile range, IQR) dosage to the parotid was 23.95 (6.14) Gy and 61.55 (20.14) Gy for the submandibular glands. Patients had completed prior radiation therapy with a median length of 2.7 years. Thirteen patients received TOMO (Helical Tomgraphy) five received VMAT (Volumetric Modulated Arc Therapy) and two received IMRT (Intensity Modulation Radiation Therapy).

**Table 1:** Demographic data of the volunteers.

	Healthy Volunteers	Head and Neck Cancer Survivors
Gender (F:M)	25 (50%): 25 (50%)	6 (30%): 14 (70%)
Age	39 ± 14	53 ± 10
Body Weight(kg)	63 ± 13	71 ± 15
Body Height (cm)	166 ± 8	166 ± 10
Body Mass Index	22.86 ± 3.74	25.60 ± 3.30
Hypertension	8 (16%)	8 (40%)
Diabetes	2 (4%)	4 (20%)
Hyperlipidemia	3 (6%)	2 (10%)
Smoking	3 (6%)	3 (15%)

Oral cancer: 5; nasopharyngeal carcinoma: 4; oropharyngeal cancer: 3; hypopharyngeal cancer: 3; parotid cancer: 2; submandibular cancer: 1; laryngeal cancer: 1; other: 1.

For 100 parotid and submandibular glands, the median dimensions and SWV are shown in Table 2. The median of the parotid glands measured 36.15 (IQR: 12.45) mm in the anterior-posterior median length. The median paramandibular depth dimension of the parotid parenchyma measured 16.95 (4.00) mm in the transverse section, and the cranio-caudal height measured 43.60 (10.10) mm. In the submandibular glands, we found an anterior-posterior length of 34.55 (IQR: 6.95) mm, a median depth of 16.60 (3.85) mm and a cranio-caudal height of 23.40 (15.30) mm. Further analysis revealed that the dimensions of the parotid and submandibular glands were related to the gender and BW (Tables 3 and 4). For parotid paramandibular depth, non-smokers (17.15 versus 13.00 mm, p < 0.01) had a larger dimension than that in smokers. Otherwise, the dimensions did not differ with age, hypertension or hyperlipidemia.

**Table 2:** Median and interquartile (IQR) of the dimensions and SWV for healthy volunteers compared to post RT salivary glands.

	Healthy Volunteers	Head and Neck Cancer Survivors	p-values
**Parotid gland**	N = 100	N = 36	
Anteroposterior length (mm)	36.15 (12.45)	39.35 (13.05)	0.25
Paramandibular depth (mm)	16.95 (4.00)	16.55 (4.70)	0.90
Cranio-caudal height (mm)	43.60 (10.10)	42.95 (10.00)	0.75
Central SWV(m/s)	1.99 (0.57)	2.43 (0.94)	<0.01^*^
**Submandibular glands**	N = 100	N = 37	
Anteroposterior length (mm)	34.55 (6.95)	30.40 (7.30)	<0.01^*^
Paramandibular depth (mm)	16.60 (3.85)	12.05 (2.70)	<0.01^*^
Cranio-caudal height (mm)	23.40(15.30)	22.00(13.80)	0.06
Central SWV(m/s)	2.32 (0.45)	2.50 (0.62)	0.04*

*<0.05.

**Table 3:** Median and interquartile (IQR) range of the dimensions and stiffness in parotid dimensions by demographic factors among healthy volunteers.

Parotid	Anteroposterior length (mm) median (IQR)	p	Paramandibular depth (mm) median (IQR)	p	Cranio-caudal height (mm) median (IQR)	p	SWV(m/s) median (IQR)	p
Gender (F:M)		0.01*		0.26		<0.01*		0.51
Female	32.05 (16.4)		16.65 (3.90)		40.05 (9.80)		2.04 (0.69)	
Male	38.90 (10.2)		17.35 (3.90)		46.00 (11.00)		1.97 (0.42)	
Age		0.89		0.51		0.66		0.27
≥40	36.95 (18.75)		17.30 (4.80)		41.60 (15.10)		2.04 (0.655)	
<40	35.55 (11.25)		16.85 (3.70)		43.95 (9.50)		1.97 (0.52)	
Body weight (kg)		<0.01*		0.02*		<0.01*		0.95
≥60	40.55 (9.50)		17.50 (4.10)		46.00 (11.20)		1.99 (0.52)	
<60	31.00 (12.30)		16.15 (4.60)		39.55 (9.70)		2.00 (0.58)	
Body mass index		0.01*		0.24		0.42		0.03*
≥23	39.95 (11.95)		17.50 (3.70)		44.20 (12.90)		2.08 (0.45)	
<23	34.05 (15.70)		16.65 (4.40)		42.15 (9.65)		1.90 (0.63)	
Hypertension		0.63		0.18		0.25		0.12
yes	36.90 (23.15)		16.10 (4.70)		38.95 (12.05)		2.155 (0.67)	
no	35.55 (11.30)		16.90 (3.90)		43.70 (9.80)		1.95 (0.51)	
Smoking		0.85		<0.01*		0.79		0.16
yes	38.90 (36.55)		13.00 (12.97)		47.15 (44.66)		2.10 (0.30)	
no	35.55 (12.45)		17.15 (3.90)		42.80 (9.50)		1.97 (0.55)	
Hyperlipidemia		0.66		0.12		0.63		0.82
yes	41.40 (30.80)		13.95 (5.40)		46.65 (18.00)		1.99 (1.03)	
no	35.55 (12.10)		16.95 (3.85)		42.80 (9.50)		1.98 (0.54)	

p-value in M-W U tests.

* < 0.05.

**Table 4:** Median and interquartile (IQR) range of the dimensions and stiffness in submandibular dimensions by demographic factors among healthy volunteers.

Submandibular	Anteroposterior length (mm) median (IQR)	p	Paramandibular depth (mm) median (IQR)	p	Cranio-caudal height (mm) median (IQR)	p	SWV(m/s) median (IQR)	p
Gender (F:M)		<0.01*		<0.01*		<0.01*		0.02*
Female	32.10 (8.60)		15.55 (3.10)		17.00 (14.50)		2.39 (0.36)	
Male	35.35 (5.40)		17.55 (2.80)		27.90 (13.00)		2.22 (0.45)	
Age		0.69		0.25		0.12		0.27
≥40	34.40 (10.90)		16.45 (3.55)		20.70 (13.30)		2.28 (0.43)	
<40	34.55 (6.05)		16.90 (3.65)		29.15 (16.60)		2.32 (0.41)	
Body Weight (kg)		<0.01*		<0.01*		0.01*		0.50
≥60	35.70 (6.40)		17.55 (2.80)		26.90 (13.40)		2.32 (0.37)	
<60	31.95 (6.90)		15.30 (3.20)		17.45 (14.40)		2.28 (0.50)	
Body mass index		0.28		0.24		0.16		0.81
≥23	33.70 (6.40)		16.30 (3.40)		27.10 (15.40)		2.31 (0.35)	
<23	35.00 (8.50)		16.80 (3.20)		19.50 (13.90)		2.34 (0.49)	
Hypertension		0.23		0.07		0.76		0.13
yes	33.15 (11.55)		15.45 (3.55)		28.60 (18.40)		2.26 (0.47)	
no	34.80 (6.30)		16.85 (3.60)		21.70 (13.50)		2.34 (0.44)	
Smoking		0.72		0.60		0.78		0.33
yes	37.70 (37.22)		16.50 (3.55)		21.70 (14.90)		2.43 (0.38)	
no	34.40 (6.80)		18.95 (17.42)		30.55 (30.30)		2.31 (0.46)	
Hyperlipidemia		0.80		0.20		0.28		0.78
yes	36.25 (12.80)		13.95 (5.20)		16.10 (17.40)		2.42 (0.41)	
no	34.55 (6.80)		16.75 (3.55)		23.40 (15.30)		2.31 (0.45)	

p-value in M-W U tests.

* < 0.05.

The dimension of the parotid glands was not significantly correlated with prior radiation; however, the SWV was significantly increased (1.99 m/s versus 2.43 m/s, p-value < 0.01). The dimension of the submandibular glands was significantly correlated with prior radiation, and the SWV was also significantly increased, 2.32 m/s versus 2.50 m/s (p-values < 0.01).

## Discussion

It is important to know the normal range of salivary gland size for reference because inflammatory disease and neoplastic condition would lead to enlargement of salivary glands, and chronic sclerosing sialoadenitis leads to atrophy of the glands^21^. This is the first study to measure both the dimensions and hardness of Asians; we found that the sizes of the parotid and submandibular glands are smaller than in a previous report with European subjects^8^. This may be because of the overall smaller body weight in our study group. Therefore, both glands were proportionally reduced in each dimension. In our study, we noticed that the dimensions of both the parotid and submandibular glands were correlated to body weight and gender. Females have smaller body weight, which may lead to smaller dimensions of the major salivary glands. In contrast to a previous study^8^, we noticed that the dimensions of SM glands were also correlated to body weight.

We also found that the dimensions of submandibular glands were smaller for post-radiation glands. However, the dimensions of parotid glands were not significantly different; which may be because of the relatively low radiation dose to the parotid glands. Additionally, in modern irradiation methods with varying intensity such as TOMO and VMAT, modifying radiation strategies could decrease the radiation dose to the parotid glands^22,23^. This is consistent with the observations in our patients, where median radiation dose to the submandibular glands was significantly higher than that to the parotid glands. Furthermore, for parotid glandular malignancy, total parotidectomy was done without residual glands; therefore, no parotid glands could be assessed.

Although the size was not significantly different, we still noticed that the SWV in a post-radiation parotid gland was higher than that in healthy volunteers. The radiation may still lead to tissue fibrosis in parotid glands first, although the size does not significantly change. Because the completion of radiation therapy occurs a median of 2.7 years earlier, the fibrotic process may need more time and may make the glands atrophy.

For submandibular glands, the size of submandibular glands was smaller for post-radiation glands and the SWV was also higher. For head and neck cancer, the radiation field often involves the submandibular glands in the level I area, and because of the higher dose of radiation for submandibular glands, the fibrosis may be more severe, leading to decreased size and increased hardness compared with non-irradiated glands. Our findings, comparable to a previous study^24^, revealed that high-resolution ultrasound and shear wave elastography may be a useful tool for salivary gland evaluation during radiation and post-treatment follow-up.

A previous study revealed that smoking would lead to decreased saliva flow and quality. However, no study has reported the relation of smoking and salivary gland dimensions. Our study found a relation between smoking and salivary gland dimension. Non-smokers have a larger parotid paramandibular depth dimension than smokers (Table 3). This finding can partially explain the reason for decreased saliva in smokers^11^.

There were some limitations in this study. We noticed that the SWV was higher in our study when compared with measurement results in other studies^17,20^. This may be because we measured the central part of the glands. Second, theoretically, the size of major salivary glands may be associated with hypertension, yet we did not find this phenomenon. This may be because the case number in this study is not robust enough, potentially leading to a null significant difference of those potential relating factors, such as hypertension and diabetes. Thus, a larger cohort study might be performed to determine the relation of various factors on size and elastography of the major salivary glands in the future. Third, a previous report revealed a mismatched volume measurement by US and cadaveric study^8^. We did not use a mathematic formula to calculate the volume of salivary glands. Fourth, theoretically, the dimensions of major salivary glands will decrease with age. However, multiple studies^7–9^, including ours, did not find an aging effect on the size of major salivary glands with ultrasound. Further studies are still necessary to clarify this inconsistency. Fifth, the procedure of ultrasound was dependent on the operator and might result in some observer bias. Therefore considerable intra-observer and inter-observer variability existed in elastography measurement and were reported in breast lesions^25^, circumscribed objects^26^, and potentially in our study. In order to avoid inter-observer variation, in the current study, the US procedure was performed by one physician and the images were interpreted by the same examiner, while inter-observer reproducibility for liver and spleen was reported to be excellent^27,28^.

We find that ultrasound is a useful tool for assessment of the dimensions and hardness of major salivary glands. The dimensions and hardness of the glands are associated with body weight and previous irradiation. This study proves that the salivary glands in an Asian population have smaller measurements than those in a European population. This study also confirms that, in this population, there is a relationship between body weight and gland size. In addition to altering the gland size, previous radiation for two or more years is shown to be correlated with smaller gland size and more fibrosis; this is confirmed with elastography.
